# Risk Factors for Complications and 90-Day Mortality After Percutaneous Endoscopic Gastrostomy: The Role of Nutritional and Inflammatory Markers

**DOI:** 10.3390/medicina61111916

**Published:** 2025-10-25

**Authors:** Nermin Mutlu Bilgiç, Güldan Kahveci, Hüseyin Aykut, Yasemin Özer, Ekmel Burak Özşenel, Sema Basat

**Affiliations:** 1 Department of Gastroenterology, Ümraniye Training and Research Hospital, University of Health Sciences, Istanbul 34000, Türkiye; huseyin.ayk@gmail.com; 2 Department of Nutritional Nursing, Ümraniye Training and Research Hospital, University of Health Sciences, Istanbul 34000, Türkiye; nurse.guldan@gmail.com; 3 Department of Internal Medicine, Ümraniye Training and Research Hospital, University of Health Sciences, Istanbul 34000, Türkiye; ysmnozer89@gmail.com (Y.Ö.); ekmelburak@gmail.com (E.B.Ö.); semaucak@hotmail.com (S.B.)

**Keywords:** percutaneous endoscopic gastrostomy, complications, mortality, albumin, C-reactive protein, prognostic nutritional index, NRS-2002, nutritional risk, inflammation, risk stratification

## Abstract

*Background and Objectives*: Percutaneous endoscopic gastrostomy (PEG) is a widely accepted method for long-term enteral nutrition, but procedure-related complications and early mortality remain major concerns. Nutritional and inflammatory indices such as serum albumin, C-reactive protein (CRP), Prognostic Nutritional Index (PNI), and Nutrition Risk Screening (NRS-2002) may provide prognostic value, yet comparative data in PEG cohorts are limited. This study aimed to identify predictors of complications and 90-day mortality after PEG and to compare the prognostic performance of nutritional indices. *Materials and Methods*: A retrospective cohort of 122 consecutive adult patients undergoing PEG between January and December 2024 was analyzed. Demographic, clinical, and laboratory parameters were collected, including albumin, CRP, PNI, and NRS-2002. Complications were categorized as early (≤30 days) or late (>30 days), and all-cause mortality was assessed at 30 and 90 days. Univariate and multivariate logistic regression models were used to evaluate predictors of complications and 90-day mortality. To address multicollinearity, albumin, PNI, and NRS-2002 were separately tested in adjusted models, with model performance assessed by AIC, BIC, Nagelkerke R^2^, and C-index. *Results*: Early complications occurred in 4.9% and late complications in 8.2% of patients, for a total complication rate of 13.1%. Thirty-day mortality was 4.1%, 90-day mortality 17.2%, and total in-hospital mortality during the study year 30.3%. Neuromuscular indication was independently associated with increased risk of complications (aOR 5.0, 95% CI 1.2–20.0, *p* = 0.028) but reduced 90-day mortality (aOR 0.15, 95% CI 0.03–0.80, *p* = 0.025). Lower baseline albumin independently predicted higher 90-day mortality (aOR 0.92, 95% CI 0.86–0.99, *p* = 0.034). Elevated CRP demonstrated a borderline association with mortality (*p* = 0.051), while NRS-2002 ≥5 and Δ-PNI showed borderline trends toward increased mortality risk. In model comparison, none of the nutritional indices achieved independent statistical significance, but all demonstrated similar performance (AIC = 114, C-index 0.72–0.74). *Conclusions*: PEG outcomes are strongly influenced by baseline indication and nutritional–inflammatory status. Neuromuscular patients and patients with dysphagia face higher complication risk but lower short-term mortality, while hypoalbuminemia, elevated CRP, and high NRS-2002 or declining PNI identify patients at greater risk of death. Systematic integration of albumin, CRP, PNI, and NRS-2002 may improve risk stratification and management in PEG candidates.

## 1. Introduction

Percutaneous endoscopic gastrostomy (PEG) provides long-term enteral nutrition for patients with impaired oral intake owing to neurologic, neuromuscular, or structural impairments. Although PEG reduces risks associated with nasogastric feeding and facilitates nutritional support, outcomes such as complications and short- to mid-term mortality remain significant concerns, particularly in frail populations [1,2].

Recent studies have underscored the role of nutritional and inflammatory biomarkers in predicting mortality among PEG recipients; lower Prognostic Nutritional Index (PNI) and higher CRP-to-albumin ratio (CAR) have been reported to be associated with increased mortality, with PNI shown to be a stronger independent predictor of one-year mortality [3,4]. Similarly, in older Japanese patients with dysphagia, severe malnutrition as classified by PNI was significantly associated with increased mortality [5].

Complications after PEG, particularly in neurologic and neuromuscular populations, are frequently reported, and elevate morbidity and healthcare burden. A Turkish cohort reported that higher CRP, low total serum protein, and albumin levels were associated with 90-day mortality [2]. Other cohorts have identified the CRP-to-albumin ratio as independent predictor of both complications and early mortality following PEG insertion [6].

Despite growing evidence, direct comparisons among nutritional indices as albumin, PNI, and NRS-2002 in PEG cohorts remain limited. Standard clinical practice often relies on albumin alone, which fails to capture immune status and inflammatory burden. Thus, rigorous multivariate analyses that include PNI and NRS-2002, as well as dynamic changes, are needed to identify independent predictors of both complications and 90-day mortality.

The present study was undertaken to evaluate predictors of complications and 90-day mortality after PEG in a single-center cohort. We specifically compare baseline and dynamic values of albumin, CRP, PNI, and NRS-2002, and assess models of prognostic performance to determine which nutritional or inflammatory index offers superior risk stratification.

## 2. Material and Methods

### 2.1. Study Design and Population

This retrospective observational study included 122 consecutive adult patients who underwent PEG between 1 January and 30 December 2024. Patient records from this period were retrospectively reviewed. Ethical approval was obtained prior to data analysis from the Ethics Committee of the University of Health Sciences, Ümraniye Training and Research Hospital (Decision No: 526; Date: 16 January 2025). All PEG procedures were performed using the pull-through technique. Patients were eligible if they were ≥18 years of age and had complete baseline laboratory data. Those with missing core clinical data or incomplete follow-up information were excluded. Patients with missing ≥30-day laboratory values were not excluded from baseline analyses but were included in subgroup analyses when follow-up data were available. Δ-values were calculated only for patients with paired baseline and follow-up results.

### 2.2. Data Collection

Demographic variables (age, sex), clinical indications for PEG (tracheostomy, Alzheimer’s disease/dementia, neuromuscular disorders, prolonged intubation, cerebrovascular accident), and comorbidities (hypertension, diabetes mellitus, dementia, cerebrovascular disease, coronary artery disease, Parkinson’s disease) were extracted from medical records.

Nutritional status was assessed using the Nutritional Risk Screening 2002 (NRS-2002). Laboratory parameters were recorded within 72 h prior to PEG placement and at ≥30 days of follow-up when available, including serum albumin, C-reactive protein (CRP), and the Prognostic Nutritional Index (PNI), calculated as: PNI = 10 × serum albumin (g/dL) + 0.005 × absolute lymphocyte count (/mm^3^).

Δ-values were calculated as follow-up minus baseline values for albumin, CRP, and PNI.

All patients had NRS-2002 ≥3; therefore, a low-risk (<3) reference group was not available. For exploratory analysis, patients were stratified as NRS 3–4 vs. ≥5.

### 2.3. Outcomes

The primary outcomes were the occurrence of any PEG-related complication (early ≤30 days or late >30 days) and 90-day all-cause mortality. Early complications included peristomal leakage, local infection, accidental dislodgement, and tube obstruction. Late complications included tube blockage, accidental cutting or dislodgement, buried bumper syndrome, hypergranulation, and other mechanical events. Mortality was assessed at 30 days, 90 days, and throughout the index hospitalization.

### 2.4. Statistical Analysis

Continuous variables were expressed as mean ± standard deviation (SD) or median (interquartile range, IQR) depending on distribution assessed by the Shapiro–Wilk test. Categorical variables were summarized as counts and percentages. Between-group comparisons were performed using the independent *t*-test or Mann–Whitney U test for continuous variables, and Chi-square or Fisher’s exact test for categorical variables.

Univariate logistic regression analyses were conducted to identify predictors of total complications and 90-day mortality. Variables with *p* < 0.10 in univariate analysis and those of established clinical relevance were entered into multivariate logistic regression models. Odds ratios (OR) and adjusted odds ratios (aOR) were reported with 95% confidence intervals (CI). To address multicollinearity between albumin, PNI, and NRS-2002, each nutritional index was tested in separate multivariate models adjusted for age, sex, PEG indication, and CRP. Model performance was evaluated using Akaike Information Criterion (AIC), Bayesian Information Criterion (BIC), Nagelkerke R^2^, and concordance statistic (C-index). A two-sided *p* < 0.05 was considered statistically significant, and values between 0.05 and 0.10 were interpreted as trend toward association. All analyses were performed using IBM SPSS Statistics version 28.0 (IBM Corp., Armonk, NY, USA).

## 3. Results

### 3.1. Baseline Characteristics

A total of 122 patients were included. The median age was 73 years (IQR 66–82; range, 25–95 years), and 48.4% were male. Tracheostomy (44.3%) and Alzheimer’s disease/dementia (30.3%) were the most frequent PEG indications, while neuromuscular disorders accounted for 15.6%. More than 90% of patients had at least one comorbidity, most commonly hypertension (54.1%) and diabetes mellitus (33.6%). The median pre-PEG albumin was 27.1 g/dL, CRP 62 mg/dL, and mean PNI 36.8 ± 7.5. At ≥30-day follow-up, albumin increased to 31.2 g/dL, CRP decreased to 28 mg/dL, and PNI improved to 39.5 (Table 1).

### 3.2. Complications and Mortality

PEG was most frequently performed in ICU patients (49.2%). Early complications occurred in 6 patients (4.9%), and late complications in 10 patients (8.2%), yielding a total complication rate of 13.1%. Thirty-day mortality was 4.1% (n = 5), 90-day mortality 17.2% (n = 21), and in-hospital total in-hospital mortality during the study year 30.3% (n = 37) (Table 2).

### 3.3. Univariate Regression Analysis

In univariate analysis, neuromuscular indication was associated with a higher risk of complications (OR 3.0, 95% CI 0.9–9.6, *p* = 0.045). Elevated baseline CRP showed a borderline association with complications (*p* = 0.050).

For 90-day mortality, low baseline albumin (OR 0.93, 95% CI 0.88–0.98, *p* = 0.005), elevated CRP (OR 1.007, 95% CI 1.002–1.012, *p* = 0.028), and low PNI (OR 0.90, 95% CI 0.84–0.97, *p* = 0.005) were significant predictors. High nutritional risk by NRS-2002 (≥5) demonstrated a borderline trend (OR 2.40, 95% CI 0.94–6.14, *p* = 0.057). Δ-PNI also showed a borderline trend (*p* = 0.053). Compared with tracheostomy, Alzheimer’s/dementia (OR 0.15, *p* = 0.001) and neuromuscular disease (OR 0.06, *p* = 0.006) were protective against 90-day mortality (Table 3).

### 3.4. Multivariate Regression Analysis

In the multivariate model for complications, neuromuscular indication remained an independent predictor (aOR 5.0, 95% CI 1.2–20.0, *p* = 0.028). Age demonstrated a borderline protective trend (aOR 0.95 per year, *p* = 0.051).

For 90-day mortality, higher baseline albumin was independently protective (aOR 0.92, 95% CI 0.86–0.99, *p* = 0.034). Neuromuscular indication was strongly protective compared with tracheostomy (aOR 0.15, 95% CI 0.03–0.80, *p* = 0.025). Baseline CRP showed a borderline association with increased mortality (aOR 1.006, 95% CI 1.000–1.012, *p* = 0.051) (Table 4).

### 3.5. Model Comparison of Nutritional Indices

To address potential multicollinearity, albumin, PNI, and NRS-2002 were tested separately in multivariate models adjusted for age, sex, PEG indication, and CRP. None of the indices reached statistical significance (albumin *p* = 0.38; PNI *p* = 0.36; NRS-2002 *p* = 0.85). All three models demonstrated similar performance: AIC ≈ 114, BIC ≈ 133, Nagelkerke R^2^ ≈ 0.20, and C-index 0.72–0.74. The apparent loss of significance for albumin (*p* = 0.034 in Table 4 vs. *p* = 0.38 in Table 5) reflects the smaller subset (N = 82) with complete follow-up data, resulting in reduced statistical power rather than a true change in effect (Table 5).

## 4. Discussion

Our analysis reveals a clear divergence in outcomes based on PEG indication. Patients with neurologic or neuromuscular disorders had higher rates of complications yet lower 90-day mortality compared to those with other indications. This suggests that although neurologically impaired patients are prone to complications (aspiration pneumonia, peristomal infection), they do not face the near-term fatal course seen in many advanced diseases patients. Although malignancy was not an indication in our cohort, prior studies confirm that PEG for malignancy carries significantly higher early mortality than PEG for neurological causes [7]. Notably, the multicenter study by Stenberg et al. found no difference in complication rates between these groups, implying the excess mortality in cancer patients stems from their illness rather than the procedure. In our cohort, however, neuromuscular patients did experience more complications, likely reflecting their vulnerability to aspiration and other care-related issues [7]. Aspiration pneumonia is a well-documented complication in PEG patients with neurologic dysphagia [7]. Neuromuscular patients frequently experience aspiration or peristomal complications due to secretion management difficulties; however, unlike malignancy or end-stage comorbidity groups, their baseline systemic condition is relatively stable, explaining the lower short-term mortality despite a higher complication rate. These findings underscore the need for tailored management: neurologic patients require vigilant prevention of complications, whereas oncology patients warrant careful prognostication due to their higher inherent mortality risk.

Several nutritional and inflammatory factors emerged as predictors or trends for 90-day mortality. Lower baseline albumin was associated with increased 90-day mortality, consistent with prior PEG literature, where hypoalbuminemia is a well-known marker of poor prognosis after PEG [2]. Beyond our findings, multiple large-scale studies have consistently confirmed that hypoalbuminemia is associated with increased early and mid-term mortality after PEG. For instance, a recent multicenter study from Europe including over 1000 patients identified albumin <30 g/dL as one of the strongest predictors of 30-day mortality [7]. Likewise, the PNI, which integrates albumin and lymphocyte count, has been validated as a robust marker of nutritional and immune status in PEG cohorts and other clinical populations [4,8]. These converging data strengthen the interpretation that both malnutrition and impaired systemic immunity contribute substantially to adverse outcomes. The borderline inverse association of age likely reflects indication confounding, since older patients were predominantly in tracheostomy or dementia groups, which exhibited lower complication rates.

Inflammatory markers have also gained prominence as prognostic tools. Elevated CRP and the CAR have been shown to predict early mortality and complication risk in PEG recipients [6,9]. Our finding of a borderline association between CRP and 90-day mortality supports these observations, suggesting that systemic inflammation may have a clinically relevant impact even when statistical significance is narrowly missed. Similarly, NRS-2002, a validated screening tool for malnutrition, has been reported to correlate with short-term mortality in palliative and hospitalized patients [10,11]. The trend toward increased mortality in patients with NRS-2002 ≥5 in our cohort parallels these reports and indicates that comprehensive nutritional screening remains essential in PEG candidates.

Elevated CRP showed a borderline association with higher mortality, reinforcing that even modest inflammation is clinically relevant. This aligns with studies finding that elevated CRP at PEG placement predicts increased early mortality [7,12]—for example, a CRP ≥5 mg/dL was an independent predictor of 30-day mortality in one cohort [12]. Similarly, patients with a high NRS-2002 tended toward higher 90-day mortality in our study, mirroring reports that severely malnourished patients (high NRS-2002) have greater short-term mortality risks [13]. Likewise, a decline in Prognostic Nutritional Index (Δ-PNI) showed a borderline link to mortality. Given that a higher baseline PNI is associated with better PEG outcomers [4,8], a post-PEG drop in PNI likely signals deteriorating nutritional status and portends worse survival. In sum, even near-significant factors (CRP, NRS-2002, Δ-PNI) all trended in a direction consistent with the principle that inflammation and malnutrition predispose to higher mortality.

Overall, our findings accord with contemporary PEG studies. Recent series report higher mortality in PEG patients with malignancy versus neurologic indications and consistently implicate malnutrition and inflammation in poor outcomes [7]. Lower baseline albumin was associated with increased 90-day mortality, consistent with prior PEG literature. In terms of complications, an elevated pre-PEG CRP-to-albumin ratio was shown to nearly triple the odds of complications in one study, consistent with the high complication rate we observed in neurologic patients, driven largely by aspiration events [14]. These comparisons reinforce that both the underlying PEG indication and a patient’s nutritional–inflammatory state are critical determinants of outcome, aligning our study with the emerging evidence in this field. Pathophysiologically, malnutrition and systemic inflammation contribute to adverse PEG outcomes through impaired immune competence, inflammatory catabolism, delayed wound healing, and increased infection susceptibility. These mechanisms may underlie the observed relationship between low albumin, elevated CRP, and increased mortality risk.

### Study Limitations

This single-center, retrospective analysis is subject to selection bias and residual confounding despite multivariable adjustment. Event counts were low for some endpoints, limiting statistical power, model stability, and the number of covariates that could be included. Follow-up laboratories (≥30-day albumin, CRP, PNI) were not available in all patients, introducing potential attrition bias and restricting Δ-parameter analyses to a subset. Indication mix was dominated by tracheostomy and neuromuscular disorders; therefore, comparisons with other indications (malignancy) cannot be inferred from these data. Finally, external validity may be limited given local practice patterns and the absence of standardized adjudication for minor complications.

## 5. Conclusions

In this cohort of patients undergoing PEG, neuromuscular indications were associated with higher complication rates. Nutritional and inflammatory markers were closely linked to outcomes: low baseline albumin independently predicted increased mortality, while elevated CRP showed a borderline trend toward higher risk. PNI and NRS-2002 demonstrated similar prognostic performance, with Δ-PNI and high NRS-2002 values trending toward worse survival. These findings emphasize that both underlying indication and nutritional–inflammatory status are critical determinants of prognosis after PEG and should be systematically considered in patient selection and peri-procedural management.

## Figures and Tables

**Table 1:** Baseline demographic, clinical, and laboratory characteristics of patients undergoing PEG placement.

Category	Variable	n (%)	Mean ± SD/Median (min–max)
	Male	59 (48.4)	–
	Female	63 (51.6)	–
**PEG indications**	Tracheostomy	54 (44.3)	–
	Alzheimer’s disease/Dementia	37 (30.3)	–
	Neuromuscular disease/Dysphagia	19 (15.6)	–
	Prolonged intubation	6 (4.9)	–
	Stroke (CVA)	6 (4.9)	–
**Comorbidities**	Hypertension	66 (54.1)	–
	Diabetes mellitus	41 (33.6)	–
	Dementia (including Alzheimer’s)	48 (39.3)	–
	Cerebrovascular disease	19 (15.6)	–
	Coronary artery disease	19 (15.6)	–
	Parkinson’s disease	14 (11.5)	–
	≥1 comorbidity	113 (92.6)	–
**NRS-2002 distribution**	NRS-2002 0–2	0 (0.0)	–
	NRS-2002 3–4	58 (47.5)	–
	NRS-2002 ≥5	64 (52.5)	–
**NRS-2002 groups**	NRS-2002 <3 (low risk)	0 (0.0)	–
	NRS-2002 ≥3 (high risk)	122 (100.0)	–
**Total NRS-2002**	Continuous variable	122	4.64 ± 0.93; 5.0 (3–6)
**Laboratory (pre-PEG)**	Albumin (g/dL)	–	28.0 ± 3.8; 27.1 (20–34)
	CRP (mg/dL)	–	64; 62 (1.8–406)
	PNI	122	36.8 ± 7.5; 37 (22–51)
**Laboratory (≥30-day follow-up)**	Albumin (g/dL)	–	31.2 ± 4.1; 31.0 (22–38)
	CRP (mg/dL)	–	28; 25 (2–150)
	PNI	98 †	39.5 ± 7.8; 39 (24–55)

Data are presented as mean ± standard deviation (SD) or median (min–max) depending on distribution (Shapiro–Wilk test). Pre-PEG values were measured within 72 h before the procedure. Follow-up values were obtained at ≥30 days after PEG placement (during hospitalization or outpatient/home care follow-up). † Number of patients with available ≥30-day follow-up laboratory data. PEG, percutaneous endoscopic gastrostomy; CRP, C-reactive protein; SD, standard deviation; NRS-2002, Nutritional Risk Screening 2002; CVA, cerebrovascular accident.

**Table 2:** Procedural details, complications, and mortality outcomes in patients undergoing PEG placement.

Category	Variable	n (%)
**Procedural unit**	ICU	60 (49.2)
	Palliative care ward	31 (25.4)
	Endoscopy unit	14 (11.5)
	Other	17 (13.9)
**Early complications (≤30 days)**	Peristomal leakage	2 (1.6)
	Local infection (discharge, cellulitis)	1 (0.8)
	Accidental tube dislodgement	2 (1.6)
	Tube blockage (lumen obstruction)	1 (0.8)
	**Any early complication**	6 (4.9)
**Late complications (>30 days)**	Tube blockage	4 (3.3)
	Accidental tube cutting/dislodgement	4 (3.3)
	Buried bumper syndrome	1 (0.8)
	Hypergranulation	1 (0.8)
	Other mechanical problems	0 (0.0)
	**Any late complication**	10 (8.2)
**Mortality**	30-day mortality	5 (4.1)
	90-day mortality	21 (17.2)
	**Total in-hospital mortality during the study year**	37 (30.3)

Data are presented as n (%) for categorical variables. Early complications were defined as events occurring within 30 days after PEG placement, and late complications as events occurring thereafter. Mortality was reported as 30-day, 90-day, and total in-hospital total in-hospital mortality during the study year. Other medical wards: internal medicine, neurology. PEG, percutaneous endoscopic gastrostomy; ICU, intensive care unit.

**Table 3:** Univariate logistic regression analysis for predictors of total PEG-related Complications and 90-day mortality.

Variable	Total PEG-Related Complications (n = 16)		90-Day Mortality (n = 21)	
	OR (95% CI)	*p*-Value	OR (95% CI)	*p*-Value
Age (per year increase)	0.97 (0.93–1.01)	0.1	1.01 (0.98–1.05)	0.47
Male sex (ref: female)	1.20 (0.45–3.19)	0.71	1.34 (0.60–3.02)	0.473
**PEG indication**				
Alzheimer’s/dementia (ref: tracheostomy)	1.30 (0.30–5.20)	0.721	0.15 (0.05–0.40)	0.001
Neuromuscular disease/Dysphagia (ref: tracheostomy)	3.00 (0.90–9.60)	0.045	0.06 (0.01–0.46)	0.006
Prolonged intubation (ref: tracheostomy)	1.80 (0.20–13.50)	0.578	0.94 (0.27–3.24)	0.924
Stroke (CVA) (ref: tracheostomy)	–	–	1.10 (0.30–4.06)	0.883
Hypertension	0.81 (0.29–2.26)	0.69	0.78 (0.34–1.80)	0.561
Diabetes mellitus	1.09 (0.39–3.03)	0.88	0.90 (0.40–2.05)	0.803
Dementia (comorbidity)	0.74 (0.25–2.14)	0.575	0.68 (0.27–1.71)	0.413
Cerebrovascular disease	1.32 (0.40–4.32)	0.647	1.27 (0.48–3.34)	0.628
Coronary artery disease	1.16 (0.36–3.72)	0.8	1.15 (0.42–3.19)	0.785
Parkinson’s disease	–	–	0.88 (0.28–2.79)	0.83
≥1 comorbidity (ref: none)	–	–	1.42 (0.31–6.60)	0.651
NRS-2002 ≥5 (high nutritional risk)	1.95 (0.72–5.26)	0.186	2.40 (0.94–6.14)	0.057
Albumin (g/dL, pre-PEG)	0.95 (0.88–1.03)	0.184	0.93 (0.88–0.98)	0.005
CRP (mg/dL, pre-PEG)	0.99 (0.98–1.00)	0.05	1.007 (1.002–1.012)	0.028
PNI (pre-PEG)	0.94 (0.85–1.04)	0.226	0.90 (0.84–0.97)	0.005
Δ-Albumin (follow-up—pre, g/dL)	0.93 (0.82–1.06)	0.275	1.03 (0.94–1.12)	0.504
Δ-CRP (follow-up—pre, mg/dL)	1.01 (0.99–1.02)	0.412	0.997 (0.992–1.003)	0.342
Δ-PNI (follow-up—pre)	0.98 (0.90–1.06)	0.61	1.08 (0.99–1.18)	0.053

Variables with *p* < 0.10 were considered for multivariate analysis. Borderline *p*-values (0.05–0.10) are interpreted as trends and not definitive evidence of association. Odds ratios (OR) <1 indicate a protective effect, while OR >1 indicates increased risk. “–“ denotes non-estimable results due to zero events in one group. Continuous variables (age, albumin, CRP, PNI, Δ-values) were entered as continuous predictors. Variables with borderline significance (*p* ≈ 0.05–0.10) were retained in interpretation as potential risk indicators. OR <1 represents a protective factor for survival. PEG, percutaneous endoscopic gastrostomy; OR, odds ratio; CI, confidence interval; CRP, C-reactive protein; PNI, Prognostic Nutritional Index; NRS-2002, Nutritional Risk Screening; CVA, cerebrovascular accident.

**Table 4:** Multivariate logistic regression analysis for predictors of total PEG-related Complications and 90-day mortality.

Variable		
Total PEG-Related Complications (n = 16)	aOR (95% CI)	*p*-Value
Neuromuscular disease (indication)	5.0 (1.2–20.0)	0.028
Age (per year increase)	0.95 (0.90–1.00)	0.051
NRS-2002 ≥5	2.4 (0.8–7.4)	0.11
**90-day mortality (n = 21)**		
Albumin (g/dL, pre-PEG)	0.92 (0.86–0.99)	0.034
Neuromuscular disease (indication)	0.15 (0.03–0.80)	0.025
CRP (mg/dL, pre-PEG)	1.006 (1.000–1.012)	0.051

Multivariate model included clinically relevant predictors and those approaching significance in univariate analyses (*p* < 0.10). Neuromuscular indication emerged as an independent predictor. Advanced age showed a borderline protective trend (*p* = 0.051). The model was adjusted for baseline albumin, CRP, and PEG indication (neuromuscular vs. tracheostomy). Albumin was independently protective, while neuromuscular indication reduced mortality risk. Elevated CRP demonstrated a borderline association with increased risk (*p* = 0.051). PEG, percutaneous endoscopic gastrostomy; aOR, adjusted odds ratio; CI, confidence interval; CRP, C-reactive protein; NRS-2002, Nutritional Risk Screening.

**Table 5:** Multivariate Logistic Regression Results for 90-Day Mortality (N = 82).

Nutritional Predictor	aOR (95% CI)	*p*-Value	AIC	BIC	Nagelkerke R^2^	C-Index
**Albumin**—per 1 g/dL	0.94 (0.81–1.08)	0.38	114	133.3	0.2	0.74
**PNI**—per unit	0.94 (0.82–1.07)	0.36	114	133.2	0.2	0.73
**NRS-2002 ≥5 vs. 3–4**	1.11 (0.39–3.16)	0.85	114.8	134	0.19	0.72

Multivariate logistic regression models were adjusted for age, sex, PEG indication, and baseline CRP. Each nutritional predictor (albumin, PNI, NRS-2002) was tested in a separate model to avoid multicollinearity. None of the nutritional indices reached statistical significance as independent predictors of 90-day mortality. All three models showed similar goodness-of-fit (AIC/BIC) and discriminative ability (C-index ≈ 0.72–0.74). N = 82 denotes patients with complete ≥30-day follow-up laboratory data (albumin, CRP, and PNI) used in multivariate model comparison. PEG, percutaneous endoscopic gastrostomy; aOR, adjusted odds ratio; CI, confidence interval; AIC, Akaike Information Criterion; BIC, Bayesian Information Criterion; R^2^, Nagelkerke’s pseudo-R^2^; C-index, concordance statistic; CRP, C-reactive protein; PNI, Prognostic Nutritional Index; NRS-2002, Nutritional Risk Screening 2002.

## Data Availability

The datasets generated and/or analyzed during the current study are available from the corresponding author on reasonable request.
